# Study of the ketogenic agent AC-1202 in mild to moderate Alzheimer's disease: a randomized, double-blind, placebo-controlled, multicenter trial

**DOI:** 10.1186/1743-7075-6-31

**Published:** 2009-08-10

**Authors:** Samuel T Henderson, Janet L Vogel, Linda J Barr, Fiona Garvin, Julie J Jones, Lauren C Costantini

**Affiliations:** 1 Accera, Inc., 380 Interlocken Crescent, Suite 780, Broomfield, Colorado 80021, USA

## Abstract

**Background:**

Alzheimer's disease (AD) is characterized by early and region-specific declines in cerebral glucose metabolism. Ketone bodies are produced by the body during glucose deprivation and are metabolized by the brain. An oral ketogenic compound, AC-1202, was tested in subjects with probable AD to examine if ketosis could improve cognitive performance.

**Methods:**

Daily administration of AC-1202 was evaluated in 152 subjects diagnosed with mild to moderate AD in a US-based, 90-day, randomized, double-blind, placebo-controlled, parallel-group study. Subjects were on a normal diet and continued taking approved AD medications. Primary cognitive end points were mean change from Baseline in the AD Assessment Scale-Cognitive subscale (ADAS-Cog), and global scores in the AD Cooperative Study – Clinical Global Impression of Change (ADCS-CGIC). AC-1202 was compared to Placebo in several population groups, including: intention-to-treat (ITT), per protocol, and dosage compliant groups. Results were also stratified by APOE4 carriage status (a predefined analysis based on the epsilon 4 (E4) variant of the apolipoprotein E gene). This trial was registered with ClinicalTrials.gov, registry number NCT00142805, information available at

**Results:**

AC-1202 significantly elevated a serum ketone body (β-hydroxybutyrate) 2 hours after administration when compared to Placebo. In each of the population groups, a significant difference was found between AC-1202 and Placebo in mean change from Baseline in ADAS-Cog score on Day 45: 1.9 point difference, p = 0.0235 in ITT; 2.53 point difference, p = 0.0324 in per protocol; 2.6 point difference, p = 0.0215 in dosage compliant. Among participants who did not carry the APOE4 allele (E4(-)), a significant difference was found between AC-1202 and Placebo in mean change from Baseline in ADAS-Cog score on Day 45 and Day 90. In the ITT population, E4(-) participants (N = 55) administered AC-1202 had a significant 4.77 point difference in mean change from Baseline in ADAS-Cog scores at Day 45 (p = 0.0005) and a 3.36 point difference at Day 90 (p = 0.0148) compared to Placebo. In the per protocol population, E4(-) participants receiving AC-1202 (N = 37) differed from placebo by 5.73 points at Day 45 (p = 0.0027) and by 4.39 points at Day 90 (p = 0.0143). In the dosage compliant population, E4(-) participants receiving AC-1202 differed from placebo by 6.26 points at Day 45 (p = 0.0011, N = 38) and 5.33 points at Day 90 (p = 0.0063, N = 35). Furthermore, a significant pharmacologic response was observed between serum β-hydroxybutyrate levels and change in ADAS-Cog scores in E4(-) subjects at Day 90 (p = 0.008). Adverse events occurred more frequently in AC-1202 subjects, were primarily restricted to the gastrointestinal system, and were mainly mild to moderate in severity and transient in nature.

**Conclusion:**

AC-1202 rapidly elevated serum ketone bodies in AD patients and resulted in significant differences in ADAS-Cog scores compared to the Placebo. Effects were most notable in APOE4(-) subjects who were dosage compliant.

## Background

Alzheimer's disease (AD) is a common, age-associated, progressive, neurodegenerative disease. Risk of developing the most common form of AD (sporadic or late onset) is principally linked to age, and the carriage status of the epsilon 4 (E4) variant of the apolipoprotein E gene (APOE). APOE4 behaves in a dominant, dose-dependent manner; a single copy increases risk of developing AD approximately 3 fold, while two copies increases risk approximately 10 fold (for review of APOE see [1]). Clinically, AD is characterized by progressive decline in memory and language, and pathologically by accumulation of senile plaques and neurofibrillar tangles. Another prominent feature of AD is regional cerebral hypometabolism. Early imaging studies revealed low cerebral metabolic rates of glucose use (CMRglu) in subjects with probable AD [2], and this is now recognized as a general feature of the disease [3]. The most commonly affected areas include the posterior cingulate, parietal, temporal and prefrontal regions [4]. These deficits can be detected in pre-symptomatic, at risk individuals, such as E4 carriers, as young adults. Such changes have been detected in cognitively normal subjects well before there is widespread neuronal loss or predicted plaque deposition, suggesting that low CMRglu is an early event in the disease [5].

The cause of the hypometabolism remains unclear and may be due to loss of neurons or dendritic fields. Alternatively, hypometabolism has been attributed to the action of AD-specific factors. Amyloid beta (Aβ) [6] and fragmentation of the ApoE4 protein [7] have both been implicated in dys-regulation of mitochondrial function. In addition, several authors have proposed changes in insulin signaling as a potential contributor to the development of hypometabolism [8-12]. Regardless of its cause, therapies aimed at correcting cellular metabolism may prove beneficial to the AD patient [13].

One such promising therapy is the induction of ketosis (for overview see [14]). Under normal circumstances, the main energy substrate for the brain is glucose. However, under certain situations, such as extended fasting, the liver will produce ketone bodies for use by extrahepatic tissues, including the brain. Three compounds are normally considered ketone bodies: β-hydroxybutyrate (BHB), acetoacetate (ACA) and acetone. Ketone bodies are an efficient fuel for cells [15,16].

Levels of circulating ketone bodies can be raised by adherence to a low-carbohydrate, high-fat, ketogenic diet. Ketogenic diets have been used extensively for the reduction of seizure frequency in children with refractive epilepsy [17] and have gained interest in several other neurological conditions, including amyotrophic lateral sclerosis [18], traumatic brain injury [19] and ischemia [20] (for review see [14,21,22]). In addition, several preclinical studies have suggested that induced ketosis may be beneficial in AD. For example, the toxic effects of the Aβ peptide in cultured neurons were shown to be mitigated by incubating the cells with BHB [23]. Also, a ketogenic diet reduced total Aβ40 and Aβ42 in a transgenic mouse model of AD after 38 days of feeding [24].

Alzheimer's disease patients frequently undergo changes in food preference toward sweet, carbohydrate-rich foods [25-27], which would make compliance to a ketogenic diet difficult. Therefore, AC-1202, a form of medium chain triglycerides (MCTs), was developed to safely elevate serum ketone bodies even in the presence of carbohydrate in the diet. MCTs were chosen for this study due to their safety profile [28], and long historical use in lipid malabsorption disorders and ketogenic diets [29]. Due to the unique physical properties of AC-1202, it is metabolized differently from the more common long chain triglycerides (LCT) [30]. If sufficient amounts of AC-1202 are consumed, a mild state of ketosis can be induced without modification of the diet. In a pilot study of mild to moderate AD patients, induction of ketosis by AC-1202 rapidly improved cognitive performance in subjects lacking the APOE4 allele [31].

The primary aim of the present study was to assess whether daily dosing of AC-1202 in mild to moderate AD subjects would improve cognitive performance as measured by change from Baseline in the AD Assessment Scale-Cognitive subscale (ADAS-Cog), and global score in the AD Cooperative Study – Clinical Global Impression of Change (ADCS-CGIC) after 90 days. Additional outcomes investigated included how cognitive scores were influenced by APOE4 genotype status, as our earlier acute dosing study suggested that AD patients who lacked an APOE4 allele may respond better to ketosis [31].

## Methods

### Participants

Subjects were screened for eligibility at one of 23 clinical sites based within the U.S. Eligible subjects were outpatients with a diagnosis of dementia of the Alzheimer type of mild to moderate severity according to NINCDS-ADRDA and DSMIV criteria, with a MMSE score of between 14 and 24 (inclusive) at Screen. Diagnosis of probable AD was performed by qualified physicians. A CT or MRI within 24 months prior to Screen had to show no signs of tumor, structural abnormality, or degenerative disease. Subjects were required to have a Modified Hachinski Ischemia Scale score ≤ 4.

Key exclusion criteria at Screen were: major depression as determined by a Cornell Scale for Depression in Dementia score of ≥ 13, clinically-significant hypothyroidism as determined by thyroid function assessment, clinically-significant B12 deficiency within 12 months prior to Baseline, clinically-significant renal disease or insufficiency, clinically-significant hepatic disease or insufficiency, and any type of diabetes.

Subjects receiving currently approved AD medications were eligible for enrollment in the study provided that they had been maintained on stable dosing for at least 3 months prior to enrollment, and were required to remain on stable dosing throughout the duration of the study.

### Ethics

The trial was carried out with institutional review board approval (Essex Institutional Review Board, Lebanon, NJ) and in accordance with the principles of the Declaration of Helsinki. Subjects and their caregivers provided written informed consent, which included an optional written provision for genotyping. At their discretion, participants could consent to be tested for APOE, and/or additional DNA markers. Genetic information was not shared with site personnel or study participants. All clinical site monitoring and data management procedures were carried out in accordance with FDA and ICH Good Clinical Practice Guidelines.

### Interventions

#### Investigational product

AC-1202 is a medium chain triglyceride composed of glycerin and caprylic acid (C8:0). The chemical name is 1,2,3-propanetriol trioctanoate (also known as tricaprylin or trioctanoin). The CAS registry number is 538-23-8 and molecular formula C_27_H_50_O_6 _(MW 470.69). The MCT for this study was NeoBee 895^® ^(Stepan Chemical Company). NeoBee 895 is a common food ingredient, made using glycerin from vegetable oil and fatty acids from coconut or palm kernel oil. NeoBee 895 is an MCT wherein >95% of the fatty acids are C8:0 with the remainder consisting of C6:0 and C10:0 fatty acids.

Investigational product was formulated as an emulsified spray dried powder consisting of 33% AC-1202, 64% gum Acacia (Instagum, CNI) and 2.6% syloid (244 FP, Grace Davison). Placebo was isocaloric to the active formulation, and consisted of a mixture of 51% gum acacia, 37% dextrose, 10% safflower oil and 2% syloid (prepared by The Chemins Company). Investigational product was given as a powder packaged in 30 gram sachets containing either active (equivalent to 10 grams of AC-1202) or matching Placebo.

The contents of the sachets were mixed in one 8 oz. glass of a liquid such as water, milk, or juice prior to consumption. These instructions were later amended to recommend reconstitution with a meal replacement drink (Ensure™, Abbott Laboratories, Inc) to improve product tolerability. For the first seven days of the study, subjects received one 30 gram sachet daily. On Day 8, the dose was increased to two 30 gram sachets daily (equivalent to 20 grams AC-1202), which was continued through Day 90. Daily doses were administered during breakfast, except on clinic visit days when the participants were administered investigational product at the clinic and asked to eat breakfast prior to their scheduled visit. On Day 104, a washout visit was conducted two weeks following the last product administration.

#### Apolipoprotein E Genotype

High molecular weight DNA was isolated from whole blood for determination of APOE genotype using standard techniques. Genotyping was performed using allele specific extension by TechnoSynapse Inc. (Douglas Hospital Research Center, Verdun, Quebec, Canada) as previously described [32].

### Study Visits

Participants were scheduled for five study visits: Screening, Baseline, and post-baseline Days 45, 90, and 104 (± 3 days). At Screening, subjects and caregiver provided written informed consent and were assessed for eligibility to enter the study. Screening assessments included: demographics, medical/surgical history, NINCDS-ADRDA criteria, DSM-IV criteria for dementia, Modified Hachinski Ischemia Scale, prior and concomitant medications, physical examination, height, weight, vital signs, CT scan/MRI (if not previously performed within the last 24 months), ECG, TSH, B12, BHB serum level, safety laboratory assessments, ADAS-Cog, MMSE and Cornell Scale for Depression in Dementia. Participants who qualified for the study returned for a Baseline visit (within 4 weeks of the Screening visit). Baseline assessments included: adverse events (since initiation of Screen), concomitant medications, vital signs, ADAS-Cog, ADCS-CGIC and MMSE. A blood sample was taken for serum BHB levels prior to dosing and 2 hr post-dosing. Visit 3 occurred 45 days (± 3 days) after the Baseline visit, and is referred to as Day 45. Day 45 assessments included: adverse events, concomitant medications, vital signs, ADAS-Cog, ADCS-CGIC and MMSE. A blood sample was taken for serum BHB levels prior to dosing and 2 hr post-dosing. Visit 4 occurred 90 days (± 3 days) after the Baseline visit and is referred to as Day 90. Day 90 assessments included: adverse events, concomitant medications, vital signs, ADAS-Cog, ADCS-CGIC and MMSE. A blood sample was taken for serum BHB levels prior to dosing and 2 hr post-dosing. Day 90 was the last day investigational product was administered. Visit 5 occurred 104 days (± 3 days) after the Baseline visit, and is referred to as Day 104. Day 104 assessments included: adverse events, concomitant medications, vital signs, weight, physical examination, ECG, safety labs, ADAS-Cog, ADCS-CGIC, and MMSE. A final blood sample was taken for serum BHB levels. Day 104 assessments examined performance after a two week washout period.

### Objectives

The primary aim of the present study was to assess if daily dosing of AC-1202 in mild to moderate AD patients would improve scoring on ADAS-Cog and ADCS-CGIC over a period of 90 days. A secondary outcome examined whether or not daily dosing of AC-1202 would improve scoring on MMSE. Ancillary objectives, as defined in the protocol, examined changes in ketone body levels post-dose, and whether efficacy was influenced by the carriage status of the epsilon 4 variant of the APOE gene.

### Outcomes

#### Cognitive testing

As required by the protocol, all cognitive testing was carried out by trained physicians, psychologists, nurses, social workers, and/or research coordinators under the direct supervision of the Principal Investigator.

The ADAS-Cog generally requires 30 to 45 minutes to complete, is one of the most widely used cognitive tests for anti-dementia drugs in the United States, and is frequently considered the "gold standard" in evaluating cognitive outcomes. The ADAS-Cog subscale consists of 11 tasks measuring cognitive abilities in memory, language, orientation, and praxis. The test includes seven performance items and four clinician-rated items, with a total score ranging from 0 (no impairment) to 70 (severe impairment). The memory items includes two performance items (word recall and word recognition), and one clinician rated item (remembering test instructions). In word recall, the subject is shown ten words on flash cards and then asked to recall the words in any order. In word recognition, the subject is read aloud 12 words from flash cards. The cards containing the 12 words read to the patient are then mixed with 12 new word containing cards, and all 24 cards shown to the subject. The subject is then asked to distinguish the new words from the words that were read aloud. Language items include two performance items (naming objects and following commands) and three clinician rated items (spoken language ability, word finding difficulty, and comprehension of spoken language). In naming objects, the subject is presented with real objects (such as pencil, wallet, or comb) and asked to name them. In the clinician rated language items, the clinician evaluates the subjects' overall ability to understand and communicate spoken language during the course of the test. The orientation item consists of one performance test, in which the subject is asked a series of questions related to where the subject physically is located and time and date. Praxis items include two performance items (constructional and ideational). In constructional praxis, the subject is asked to draw several specific geometric shapes. In ideational praxis, the subject is asked to perform a task, such as mailing a letter, and is scored on the ability to complete the task. The higher the ADAS-Cog score, the more impaired the subject. Lowering of the ADAS-Cog score is a measure of cognitive improvement.

The MMSE is a simple test used primarily for screening for dementia. It can be administered in 5–10 minutes. The MMSE test measures several cognitive areas including: orientation, word registration, calculation, word recall, language and visual construction. Orientation (10 points) is measured by a series of questions relating to time and place (What is the year? What is the month? What state are we in?). Word registration (3 points) is measured by reading the subject three words and then the subject is asked to repeat them. Attention and calculation (5 points) is measured by asking the subject to count backward from 100 by 7 s. Word recall (3 points) is measured by asking the subject to recall the 3 words from the word registration test. Language (8 points) is measured by asking the subject questions requiring the patient to name objects such as pencil or a wallet. Visual construction (1 point) is measured by having the subject draw a specific geometric shape. Higher scores on MMSE indicate less impairment. The MMSE is much less sensitive than the ADAS-Cog to change, yet is advantageous in that it can be easily administered.

The ADCS-CGIC is used to assess a meaningful clinical change over time. The ADCS-CGIC focuses on clinicians' observations of change in the subject's cognitive, functional and behavioral performance from Baseline status. Unlike ADAS-Cog or MMSE, CGIC takes into account a subject's overall function in the cognitive, behavioral and functional activity domains. The CGIC consists of two parts. Part I is the Baseline evaluation during which the clinician interviews the patient and the caregiver to establish a reference point for future ratings. Part II is the follow-up interview with the patient and caregiver, and is used to evaluate the overall status of the patient relative to the reference Baseline interview. The CGIC uses a seven point scale, from 1 (marked improvement) to 7 (marked worsening). Therefore, like the ADAS-Cog, lower scores indicate improved performance. (Note: due to a printing error in the original Case Report Forms (CRFs), a six-point scale was initially used that inadvertently omitted the category "minimal improvement." The scale was revised to include the full seven-point scale at a later time point.)

### Serum β-hydroxybutyrate

Pre- and post-dosing serum samples were collected and analyzed by Allied Research International (formerly SFBC) of Miami, FL using the BHB Liquicolor^® ^diagnostic kit supplied by Stanbio Laboratories (Boenre, TX). The normal range (12-hour fasting) is 0.02 mM to 0.27 mM.

### Safety analyses

Safety evaluations included physical examinations, vital sign measurements, routine serum chemistry and hematology tests, urinalyses and electrocardiograms performed at Screen and Day 104. Adverse events and any changes in concomitant medication administration were recorded at all clinic visits.

### Sample size

The targeted sample size of 100 (50 AC-1202 and 50 Placebo) was determined empirically. Due to the exploratory nature of this study and the lack of clinical/statistical references for ADAS-Cog change effect sizes associated with ketosis, the formal statistical power could not be calculated.

### Randomization

**Figure 1:**
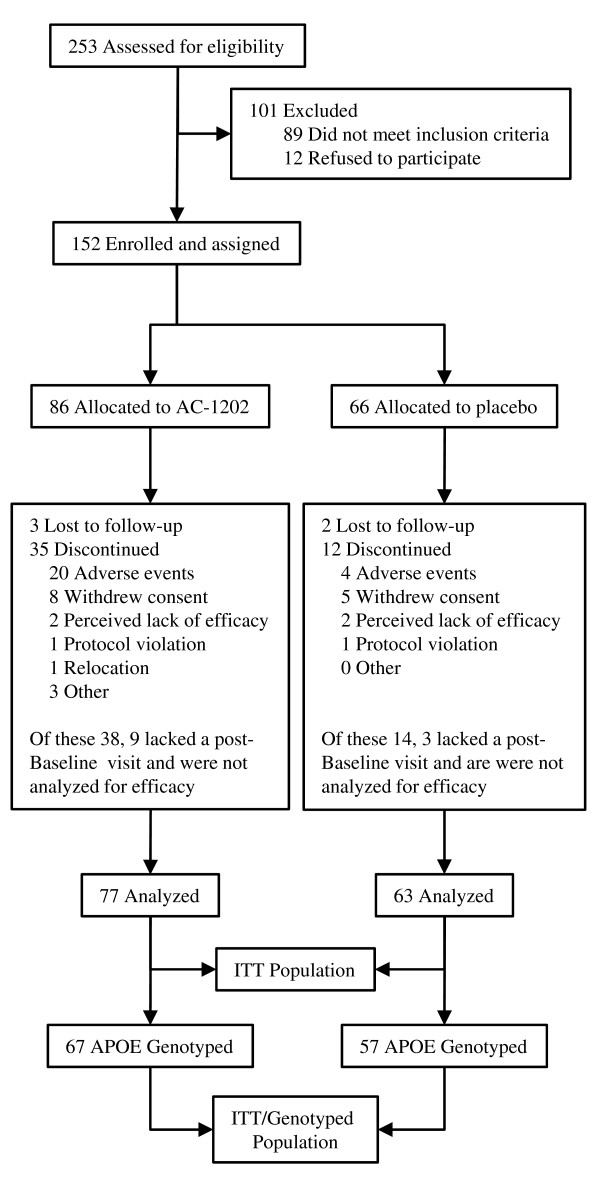
**Study randomization and group allocation for the ITT population**.

Subjects were randomized in a 1:1 ratio to receive either AC-1202 or matching Placebo for 90 days. A permutated block randomization code with a block size of 4 was used. Subjects were issued study kits labeled with a unique site and subject number. Near the end of the study, new participants entering the study were assigned to either AC-1202 or Placebo by an un-blinded independent medical monitor in such a manner as to obtain approximately 50 subjects who completed the study within each group. All clinical site personnel remained blinded throughout the entire course of the study. This intentional allocation resulted in an imbalance in the final number of randomized participants in each of the study groups; 86 randomized to AC-1202 and 66 randomized to Placebo (Figure 1). There was no crossover of participants. All subjects remained in their assigned group throughout the course of the study.

### Blinding

The participants, those administering the interventions, and those assessing the outcomes were blinded to group assignment. The double-blinding was achieved through the production and use of a Placebo similar in taste and appearance to the investigational product AC-1202.

### Statistical Methods

As defined by the protocol, an intention-to-treat (ITT) analysis was used as the primary analysis of efficacy. The ITT population was defined as all subjects who were randomized, administered at least one dose of investigational product, and completed at least one follow-up visit. For those subjects in the ITT population, missing efficacy data (i.e. ADAS-Cog, MMSE and ADCS-CGIC) was imputed using the last observation carried forward (LOCF) method. The primary end points established *a priori *were change from Baseline at Day 90 in ADAS-Cog and comparison of the global scores of ADCS-CGIC at Day 90. The secondary outcome was change from Baseline at Day 90 in the MMSE score. Pre-defined additional analyses included interactions between cognitive outcomes and APOE genotype, safety, tolerability and BHB concentration levels. Furthermore, due to the limitations of ITT w/LOCF, additional analyses were conducted on per protocol and dosage compliant populations.

An overall two-way ANCOVA was used to evaluate the cognitive scores, along with genotype effects and cognitive scores by genotype interactions for 2 hour post-dose serum BHB levels at Day 90. The ANCOVA model included independent factors for group assignment, genotype, and group assignment by genotype interactions. A variable for Baseline serum BHB level was included as a covariate. Correlations between the 2 hour serum BHB level on Day 90 and the change from Baseline ADAS-Cog total score was determined by Pearson correlation statistics.

Summary statistical analyses were provided by SIRO Inc. Mumbai, India; ANOVA and ANCOVA analyses were performed by JJo Inc. Breckenridge, CO, USA; and Accera Inc. Broomfield, CO USA.

Accera funded the study, designed the protocol, and either conducted or commissioned the data analysis and interpretation. The data are maintained on file at the offices of Accera, Inc Broomfield, CO.

## Results

### Participant flow/Numbers analyzed

Figure 1 illustrates the trial participant profile. Two hundred fifty-three participants with a diagnosis of probable AD were screened between October, 2004 and March, 2006. Of the 253 participants screened, 152 were enrolled in this study. The ITT population was defined in the protocol as all subjects who were administered at least one dose and completed at least one follow-up visit subsequent to Baseline. One hundred and forty subjects met these criteria and comprise the ITT population, of whom 77 were in the AC-1202 group and 63 in the Placebo group (Figure 1). Fifty-two subjects withdrew before Day 104; twenty-four were due to adverse events (AEs), with the majority in the AC-1202 group (Figure 1). One-hundred thirty-five subjects (n = 75 AC-1202 (AC); n = 60 Placebo (PL)) consented to genotyping for the APOE locus. Of these 135 subjects, 124 (n = 67 AC; n = 57 PL) were in the ITT population. These 124 subjects were used in the analysis of APOE4 effects.

### Recruitment

This was a randomized, double-blind, placebo-controlled, parallel-group, multi-center trial sponsored by Accera, Inc. of Broomfield, CO. It was conducted between October 5, 2004 and June 29, 2006 at 23 centers located within the United States. A list of investigator sites is found in Table 1. This was an out-patient study, and recruitment and testing was performed in individual investigators' clinical sites. All investigators in this trial were pre-qualified for study participation based on their academic training and specialty area of practice. Recruitment was done by individual investigator sites and consisted primarily of advertisements in local newspapers and on local radio stations.

**Table 1:** Investigators and sites

**Site #**	**Investigator Name**	**Institution Name, location**
100	Mildred V. Farmer, MD	Meridien Research Tampa, FL 33606 St. Petersburg, FL 33709 Brooksville, FL 34613

101	Margarita Nunez, MD	Comprehensive NeuroScience St. Petersburg, FL 33702

102	David Sack, MD	Comprehensive NeuroScience Cerritos, CA 90703

103	Murray A. Kimmel, DO	KimmelCare, Family Practice, P.A. Melbourne, FL 32935

104	Kerri L. Wilks, MD	Baumel-Eisner Neuromedical Institute Ft. Lauderdale, FL 33321 Miami Beach, FL 33154 Boca Raton, FL 33486

105	Stephen Flitman, MD	21st Century Neurology Phoenix, AZ 85013

106	Richard Hubbard, MD	The Southwest Institute for Clinical Research Rancho Mirage, CA 92270

107	Daniel E. Grosz, MD	Pharmacology Research Institute Northridge, CA 91324 Los Alamitos, CA 90720 Riverside, CA 92506 Newport Beach, CA 92660

108	Malcolm Stewart, MD	Dallas, TX 75231

109	Thomas R. Weiss, MD	Radiant Research San Antonio, TX 78229

110	Concetta Forchetti, MD	Radiant Research Chicago, IL 60610

111	Jack R. Tomlinson, MD	Grayline Clinical Drug Trials Wichita Falls, TX 76309

112	Jimmie Tarro, MD	Radiant Research Portland, OR 97239

113	Fazila Siddiqi, MD	Research Across America Dallas, TX 75234

114	Brian H. Goldman, MD	Triangle Medical Research Raleigh, NC 27609

115	Jay Rubin, MD	Renstar Medical Research Ocala, FL 34471

116	Harvey Schwartz, MD	Sunrise Clinical Research Hollywood, Fl 33021

117	Cynthia Bell, MD	Anchor Research Center Naples, FL 34102

121	James Goldenberg, MD	Visions Clinical Research Boynton Beach, FL 33437

122	David Steiner, MD	Five Towns Neuroscience Institute Cedarhurst, NY 11516

123	William Petrie, MD	Psychiatric Consultants Nashville, TN 37203

124	Joseph Soufer, MD	Phoenix Internal Medicine Associates Waterbury CT 06708

125	Michael Lesem, MD	Claghorn-Lesem Research Clinic Bellaire, TX 77401

### Baseline Data

Baseline characteristics for Placebo and AC-1202 groups are shown in Table 2. Placebo and AC-1202 groups were well matched for age, height, weight and sex. Placebo and AC-1202 groups were also comparable in terms of Baseline MMSE and ADAS-Cog scores. Sixty-six of 86 (78.7%) AC-1202 subjects and 55 of 66 Placebo subjects (83.3%) were taking currently approved AD medications. The proportion of subjects taking more than one AD medication was higher among Placebo subjects than among AC-1202 subjects (24 of 86 [27.9%] AC; 24 of 66 [36.4%] PL). Of the genotyped subjects, fifty-six percent of the subjects were E4(+) (n = 69, 55.6%), and forty-four percent were E4(-) (n = 55, 44.4%). E4(+) and E4(-) participants were not significantly different in age, sex, Baseline ADAS-Cog, or Baseline MMSE (Table 3). The proportion of subjects taking individual concomitant AD medications was similar among E4(-) and E4(+) subjects, however, more E4(+) subjects (70%) were taking memantine than E4(-) subjects (50%).

**Table 2:** Demographic Characteristics

**Safety population**		**AC-1202** **N = 86**	**Placebo** **N = 66**
Age	Mean (± SD) Median	76.9 (± 8.9) 78.0	76.8 (± 7.4) 78.0
	Range	(52 – 93)	(51 – 89)

Height (cms)	Mean (± SD)	165.2 (± 11.4)	163.2 (± 16.4)
	Median	163.8	164.6
	Range	(142.2 – 190.1)	(114.3 – 185.4)

Weight (kg)	Mean (± SD)	69.0 (± 15.15)	70.6 (± 13.7)
	Median	69.2	67.8
	Range	(34.3 – 100.2)	(47.6 – 100.2)

Sex n (%)	Male	36 (41.7)	31 (47.0)
	Female	50 (58.1)	35 (53.0)

Race n (%)	Caucasian	78 (90.7)	61 (92.4)
	Black	1 (1.2)	0
	Hispanic	7 (8.1)	5 (7.6)

Level of Education n (%)	Graduate/Professional Training	17 (19.8)	7 (10.6)
	Some college	15 (17.4)	17 (25.8)
	High School	49 (57.0)	34 (51.5)
	Grade School	5 (5.8)	8 (12.1)

AD medications n (%)*	Aricept™	43 (50)	28 (42.4)
	Exelon™	11 (12.8)	11 (16.7)
	Namenda™	32 (37.2)	31 (47)
	Reminyl™/Razadyne™	3 (3.5)	9 (13.6)

**Genotyped population**		**AC-1202** **N = 67**	**Placebo** **N = 57**

APOE Genotype n (%)	3/2	4 (6.0)	2 (3.5)
	3/3	25 (37.3)	24 (42.1)
	4/2 4/3	3 (4.9) 31 (46.3)	0 21 (36.8)
	4/4	4 (6.0)	10 (17.5)
	Total E4(+)	38 (56.7)	31 (54.4)
	Total E4(-)	29 (43.3)	26 (45.6)

**ITT population**		**AC-1202** **N = 77**	**Placebo ** **N = 63**

Baseline MMSE	Mean (± SD)	19.68 (± 4.48)	19.48 (± 4.37)
	Median	20.00	20.00
	Range	(10 – 28)	(8 – 29)
	95% CI	18.66, 20.69	18.37, 20.58

Baseline ADAS-Cog	Mean (± SD)	23.88 (± 9.17)	23.35 (± 8.7)
	Median	23.67	23.00
	Range	(7.00 – 54.33)	(11.33 – 62.00)
	95% CI	21.80, 25.96	21.16, 25.54

*Some participants were on more than one AD medication

**Table 3:** Demographic Characteristics by APOE4 status (N)

	**APOE4(-)**	**APOE4(+)**	p-value*
Mean Age	76.2 (54)	76.5 (68)	0.796

Mean ADAS-Cog	21.9 (59)	23.4 (69)	0.273

Mean MMSE	20.3 (55)	19.4 (70)	0.239

Sex	F 59% (37)	F 53% (38)	
F = female, M = male	M 41% (26)	M 47% (34)	0.603

Donepezil	N 60% (38)	N 47% (34)	
	Y 40% (25)	Y 53% (38)	0.167

Memantine	N 70% (44)	N 50% (36)	0.023
	Y 30% (19)	Y 50% (36)	

Galantamine	N 95% (60)	N 92% (66)	0.502
	Y 5% (3)	Y 8% (6)	

Number of AD medications	0 – 30% (19)	0 – 15% (11)	
	1 – 51% (32)	1 – 44% (32)	
	2 – 19% (12)	2 – 40% (29)	0.012^†^

Completed study	N 37% (23)	N 33% (24)	0.721
	Y 63% (40)	Y 67% (48)	

N = no, Y = yes

*Fisher Exact test

^†^Contingency analysis

### Extent of Exposure

One hundred fifty-two subjects were randomized in this study (86 AC; 66 PL) and received at least one dose of investigation product. Subjects were permitted to interrupt or reduce their doses of investigational product, with the permission of principal investigators, if necessitated by adverse events. Subjects in the Placebo group received higher cumulative doses and remained on-study for a longer period of time than did subjects receiving AC-1202. Over the course of the study, the AC-1202 group received a median cumulative dose of 4515 grams over a period of 90 days (representing 83.9% of the total intended dose of 5190 grams stipulated by the protocol). The Placebo group received a median cumulative dose of 4965 grams over a period of 90 days (representing 95. 7% of intended dose) (Table 4).

**Table 4:** Total cumulative dosing and treatment duration

**Safety population**	**AC-1202** **n = 86**		**Placebo** **n = 66**	
Total Cumulative Dose				
Mean dose (grams) ± SD	3555.00 ± 1883.89		4424.55 ± 1326.78	
Median	4515		4965	
Median percent of intended dose	86.99%		95.66%	
(Range)	(30, 5460)		(150, 5550)	

Treatment Duration (days)				
N*	82		66	
Mean ± SD	71.66 ± 31.08		83.91 ± 18.26	
Median	90.0		91.0	
(Range)	(1, 101)		(5, 103)	

**Genotyped population**	**APOE4(-)** **n = 36**	**APOE4(+)** **n = 39**	**APOE4(-)** **n = 27**	**APOE4(+)** **n = 33**

Total Cumulative Dose				
Mean dose (grams)	3049.17	4015.38	4382.22	4375.45
± SD	± 2076.41	± 1531.93	1398.93	± 1386.91
Median	3780	4740	4950	4980
Median percent of intended dose	72.8%	91.3%	95.4%	95.6%
Range (grams)	30 – 5430	30 – 5460	240 – 5490	150 – 5550

Treatment Duration (days)				
N*	34	38	27	33
Mean	64.1	77.8	87.1	80.3
± SD	± 36.44	± 24.3	± 11.2	+23.3
Median	88.0	90.0	91.0	91.0
Range (days)	3 – 96	1 – 101	56 – 103	5 – 100

*Treatment duration was unknown for 4 AC-1202 subjects for whom last date of study medication was not recorded

Similarly, among both APOE4(-) and APOE4(+) subjects, mean and median cumulative doses administered in the Placebo group were greater than the cumulative doses administered in the AC-1202 group. The cumulative doses received in APOE4(-) subjects were less than that for APOE4(+) subjects in the each treatment group. Median cumulative doses in APOE4(-) subjects was 3780 grams for AC-1202 subjects and 4950 grams for Placebo subjects. For APOE4(+) subjects, median cumulative dose was 4740 grams for AC-1202 subjects and 4970 grams in Placebo subjects (Table 4).

The average number of days on treatment among APOE4(-) subjects receiving AC-1202 was less than that observed among the other three cohorts. Median time on therapy for APOE4(-) subjects receiving AC-1202 was 88 days, whereas the median time on therapy for the other three cohorts ranged from 90 – 91 days (Table 4).

### Outcomes and estimations

#### ADAS-Cog

**Figure 2:**
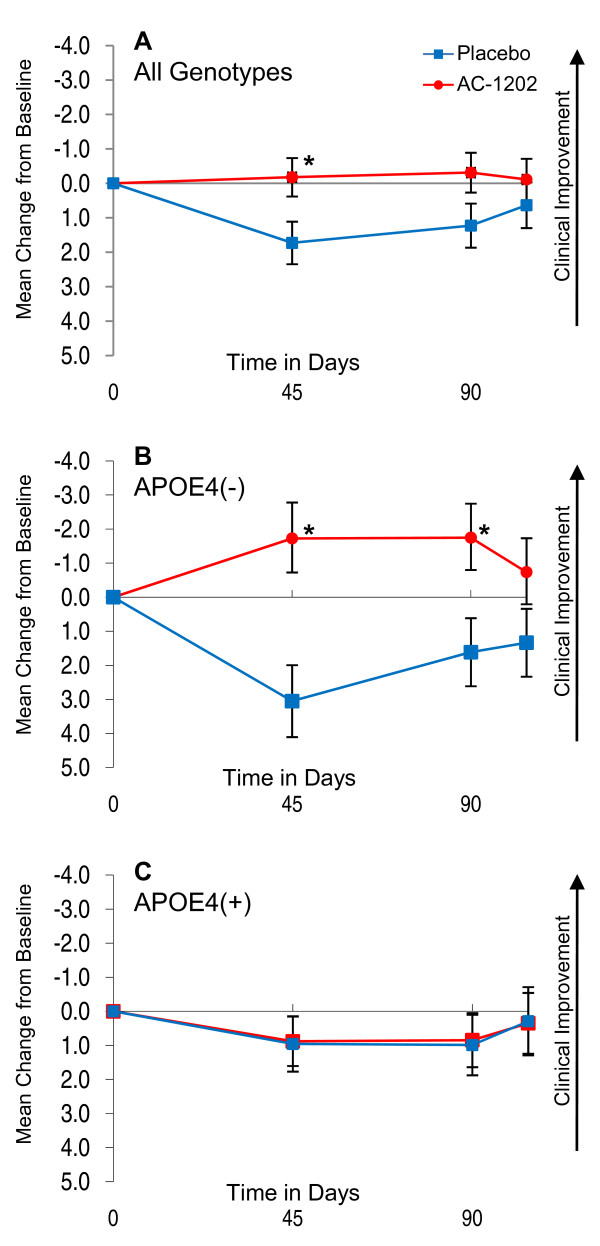
**Mean change in ADAS-Cog scores from Baseline in the ITT population w/LOCF and stratified by APOE4 carriage status**. Y axis is change from Baseline. X axis is time in days. Red circles and lines represent subjects taking AC-1202. Blue squares and lines represent subjects taking Placebo. Error bars represent standard error of the mean. Asterisks (*) indicate a significant (p-value < 0.05) difference in mean change from Baseline between AC-1202 and Placebo. **A) **Intention to treat subjects (N = 77AC, N = 63PL) administered AC-1202 demonstrate a significant difference from Placebo at Day 45. **B) **Genotyped subjects lacking the APOE4 allele (APOE4(-)) (N = 29AC, N = 26PL) and administered AC-1202 demonstrate a significant difference from Placebo at Days 45 and 90. **C) **Genotyped subjects carrying the APOE4 allele (APOE4(+)) (N = 38AC, N = 31PL) do not differ from Placebo at any time point. For confidence intervals and p-values see Table 5.

Primary outcomes defined by the protocol were change from Baseline in ADAS-Cog at Day 90 and total CGIC scores at Day 90. Figure 2A illustrates changes in ADAS-Cog scores over time in AC-1202 and Placebo ITT groups relative to Baseline. Change in ADAS-Cog scores were evaluated at Day 45, Day 90 and Day 104. Since higher ADAS-Cog scores represent increased impairment, a negative score in change from Baseline represents an improvement in cognitive performance (note Y axis is reversed in Figure 2 to illustrate the inverse relationship between improvement and ADAS-Cog scores). For the ADAS-Cog in the ITT population, the mean change from Baseline in subjects administered AC-1202 at Day 90 was -0.31 points, which was not significantly different from Placebo subjects, who increased 1.23 points (p = 0.077; see Figure 2A, Table 5). On Day 45 the mean change from Baseline in those administered AC-1202 was -0.177 points, which was significantly different from Placebo subjects, who increased 1.73 points (p = 0.024) (Figure 2A, Table 5).

**Table 5:** Efficacy by visit and genotype in the ITT w/LOCF population*

**Group; N**	**AC-1202**	**Placebo**	**Difference (95% CI)**	**p-value**
	**Day 45 Mean Change From Baseline**			

**ADAS-Cog; ITT w/LOCF**				
ITT; 77AC, 63PL	-0.177	1.730	1.91(0.26, 3.55)	0.0235
APOE4(-); 29AC, 26PL	-1.724	3.050	4.77(2.13, 7.41)	0.0005
APOE4(+); 38AC, 31PL	0.904	0.957	0.05(-2.31, 2.42)	0.9644
**MMSE; ITT w/LOCF**				
ITT; 77AC, 63PL	0.013	-0.238	-0.25(-1.12, 0.62)	0.5693
APOE4(-); 29AC, 26PL	-0.276	0.038	0.31(-1.05, 1.68)	0.6496
APOE4(+); 38AC, 31PL	-0.105	-0.645	-0.54(-1.76, 0.68)	0.3844
**CGIC^†^; ITT w/LOCF**				
ITT; 61AC, 58PL	4.21	4.43	0.22(-0.25, 0.68)	0.3536
APOE4(-); 23AC, 24PL	4.22	5.04	0.82(0.11, 1.54)	0.0240
APOE4(+); 30AC, 28PL	4.27	3.79	-0.48(-1.12, 0.16)	0.1407
**ADAS-Cog; randomized only w/LOCF**				
ITT; 61AC, 62PL	0.000	1.725	1.73(-0.04, 3.49)	0.0548
APOE4(-); 24AC, 26PL	-1.514	3.050	4.56(1.84, 7.29)	0.0012
APOE4(+); 31AC, 30PL	1.054	0.922	0.13(-2.34, 2.60)	0.9161

	**Day 90 Mean Change From Baseline**			

**ADAS-Cog; ITT w/LOCF**				
ITT; 77AC, 63PL	-0.312	1.227	1.54(-0.17, 3.24)	0.0767
APOE4(-); 29AC, 26PL	-1.747	1.614	3.36(0.67, 6.05)	0.0148
APOE4(+); 38AC, 31PL	0.868	0.989	0.12(-2.29, 2.53)	0.9211
**MMSE; ITT w/LOCF**				
ITT; 77AC, 63PL	-0.206	-0.299	0.09(-0.81, 0.99)	0.8397
APOE4(-); 29AC, 26PL	-0.276	0.385	0.66(-0.80, 2.12)	0.3710
APOE4(+); 38AC, 31PL	-0.474	-0.710	-0.24(-1.54, 1.07)	0.7209
**CGIC^†^; ITT w/LOCF**				
ITT; 64AC, 60PL	4.41	4.62	0.21(-0.29, 0.71)	0.4089
APOE4(-); 23AC, 25PL	4.17	4.68	0.51(-0.30, 1.32)	0.2180
APOE4(+); 33AC, 29PL	4.48	4.38	-0.10(-0.82, 0.61)	0.7698
**ADAS-Cog; randomized only w/LOCF**				
ITT; 61AC, 62PL	-0.093	0.908	1.00(-0.68, 2.70)	0.2420
APOE4(-); 24AC, 26PL	-1.070	1.614	2.68(0.05, 5.32)	0.0457
APOE4(+); 31AC, 30PL	1.086	0.322	0.76(-1.62, 3.14)	0.5265

	**Day 104 Mean Change from Baseline**			

**ADAS-Cog; ITT w/LOCF**				
ITT; 77AC, 63PL	-0.113	0.636	0.75(-1.02, 2.52)	0.4046
APOE4(-); 29AC, 26PL	-0.736	1.336	2.07(-0.79, 4.93)	0.1540
APOE4(+); 38AC, 31PL	0.544	0.290	-0.25(-2.82, 2.31)	0.8450
**MMSE; ITT w/LOCF**				
ITT; 77AC, 63PL	-0.351	-0.619	-0.27(-1.33, 0.79)	0.6177
APOE4(-); 29AC, 26PL	-0.586	-0.385	0.20(-1.52, 1.92)	0.8166
APOE4(+); 38AC, 31PL	-0.368	-0.968	-0.60(-2.14, 0.94)	0.4421
**CGIC^†^; ITT w/LOCF**				
ITT; 75AC, 63PL	4.65	4.81	0.16(-0.29, 0.60))	0.4915
APOE4(-); 28AC, 26PL	4.39	5.00	0.61(-0.09, 1.31)	0.0877
APOE4(+); 37AC, 31PL	4.73	4.48	-0.25(-0.87, 0.31)	0.4369
**ADAS-Cog; randomized only w/LOCF**				
ITT; 61AC, 62PL	0.022	0.431	0.41(-1.43, 2.25)	0.6597
APOE4(-); 24AC, 26PL	-0.583	1.336	1.92(-0.99, 4.83)	0.1937
APOE4(+); 31AC, 30PL	0.398	-0.144	0.54(-2.09, 3.17)	0.6838

ITT = Intention-To-Treat; AC = AC-1202; PL = Placebo.

*2 way ANOVA calculated using PROC GLM Type 3 SS. P-values of differences of the type 3 SS LSMEANS.

^†^CGIC Mean Total scores at Days 45, 90 and 104. Note, some participants did not complete a Day 45 CGIC and no value was carried forward to Day 90.

#### CGIC

For the CGIC, the mean CGIC score in subjects administered AC-1202 at Day 90 was 4.21 points, which was not significantly different from Placebo subjects, whose score was 4.43 points (p = 0.354; Table 5). The CGIC did not differ between groups at Day 45.

#### MMSE

The mean change from Baseline MMSE score in subjects administered AC-1202 at Day 90 was 0.013 points, which was not significantly different from Placebo subjects, whose change was -0.238 points (p = 0.569; Table 5).

### Ancillary analyses

Additional analyses as defined by the protocol included: ability of AC-1202 to induce ketosis, changes in test scores after the 2 week Washout, and if cognitive scores were influenced by the presence or absence of an APOE4 allele.

### Ketosis

**Figure 3:**
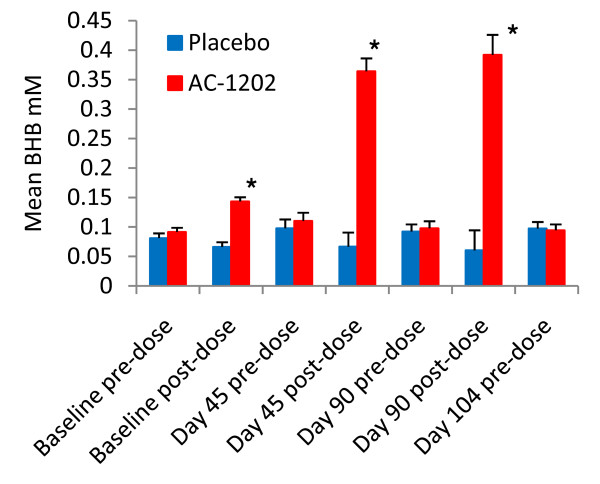
**Mean pre and post-dose serum BHB levels at each study visit**. Post-dose samples were taken 2 hours after administration of investigational product. Blue bars represent Placebo, red bars represent AC-1202. Error bars represent standard error of the mean. Significant increases in BHB levels were found post-dose in AC-1202 subjects compared to Placebo subjects. Subjects were given 1/2 dose at Baseline (10 grams), full dose on Days 45 and 90 (20 grams), and no dose was administered on Day 104. Asterisks (*) represent significant differences between AC-1202 and Placebo groups (p < 0.0001).

AC-1202 resulted in significantly elevated BHB levels at all post-dose time points. Screening BHB levels were within normal ranges and did not differ between groups (0.11 ± 0.08 mM AC; 0.12 ± 0.11 mM PL, p = 0.590, ± values indicate SEM). AC-1202 induced a significant elevation in serum BHB levels on all visit days in which investigational material was administered (Figure 3). At Baseline, subjects received 1/2 dose of AC-1202 (10 grams) and mean serum BHB increased from 0.09 mM at pre-dose, to 0.14 mM 2 hours post-dose, which was significantly different from the Placebo group (p < 0.0001). Higher post-dose levels of BHB were obtained on Day 45 and Day 90 when subjects were given a full dose (20 grams). Average post-dose BHB values in the AC-1202 group were 0.36 mM on Day 45 and 0.39 mM on Day 90, both significantly different from Placebo group (p < 0.0001). BHB levels were not different between AC-1202 and Placebo groups at any pre-dose sampling or after the 2 week Washout visit (Figure 3).

### Changes after 2 week Washout

On Day 104, after the two week Washout, there was no significant difference in ADAS-Cog or CGIC in the ITT population between groups (Figure 2A, Table 5).

### Effect of APOE4 status

A previous study had demonstrated that acute administration of AC-1202 resulted in improvement in ADAS-Cog in APOE4(-) AD patients [31]. To examine if similar effects were seen in the present study, the effects of AC-1202 administration on ADAS-Cog, MMSE and CGIC measures were stratified by APOE4 carriage status. This analysis examined the 124 subjects who provided written genetic consent for APOE testing, and used the ITT population with LOCF.

#### ADAS-Cog

Among E4(-) patients, those administered AC-1202 performed significantly better on the ADAS-Cog relative to Placebo at both Day 45 and Day 90. AC-1202 participants improved -1.72 points over Baseline at Day 45, compared to a 3.05 point decline in Placebo (p = 0.0005), and improved -1.75 points over Baseline at Day 90 compared to a 1.61 point decline in Placebo (p = 0.0148) (Figure 2B, Table 5). Furthermore, the percentage of E4(-) subjects experiencing categorical improvements in ADAS-cog scores were notably higher in the AC-1202 group than in Placebo. At Day 90, 41.4% (12/29) of E4(-) participants in the AC-1202 group experienced a -2 point or greater improvement from Baseline compared to 19% (5/26) in the Placebo group. In addition, 31% (9/29) experienced a -4 point or greater improvement compared to 7.7% (2/26) in the Placebo group, and 13.8% (4/29) experienced a -10 point or greater improvement compared to 3.8% (1/26) in the Placebo group. After Washout, among E4(-) subjects, there was no difference between AC-1202 and Placebo groups (p = 0.154). Among E4(+) subjects, there were no significant effects in changes in ADAS-Cog at any time point over the course of the study (Figure 2C, Table 5).

#### CGIC

Among E4(-) subjects, those administered AC-1202 showed significantly lower CGIC scores at Day 45 (p = 0.024) compared with Placebo (lower scores indicate improvement). CGIC scores at Day 90 and Day 104 in the AC-1202 group were not significantly different from Placebo (Day 90, p = 0.218; Day 104, p = 0.0877). Among E4(+) participants, average scores on CGIC were similar to Placebo at all time points (Table 5).

#### MMSE

No significant effects were found at any time point regardless of genotype (Table 5).

### Randomized only subjects

To examine whether the positive results in the ADAS-Cog test were due to the assignment of new subjects into groups by the un-blinded monitor (see Methods, Randomization), an analysis was completed on "randomized only" participants by excluding all subjects intentionally assigned by the independent, un-blinded monitor. Among the ITT population, 17 participants were assigned by the independent monitor; 16 to the AC-1202 and 1 to Placebo, leaving 61 randomly assigned to AC-1202 and 62 to Placebo. Among the "randomized only" population, there was no difference in change from Baseline in ADAS-Cog scores between those administered AC-1202 compared to those administered Placebo on Day 45 (p = 0.0548), Day 90 (p = 0.242), or Day 104 (p = 0.660). Among E4(-) "randomized only" subjects, those taking AC-1202 performed significantly better on the ADAS-Cog relative to Placebo at both Day 45 (4.56 point difference; p = 0.00123) and at Day 90 (2.68 point difference; p = 0.0457) when compared to Baseline scores (Table 5).

### Per protocol subjects

To examine if any bias was present due to the relatively high dropout rate in the AC-1202 group and the use of LOCF, an analysis was completed on "per protocol" subjects. Per protocol subjects were defined as subjects who completed all ADAS-Cog, MMSE and CGIC measures for whom no data were carried forward. All data for per protocol subjects represent actual scores on that study visit and no data were imputed.

#### ADAS-Cog

**Figure 4:**
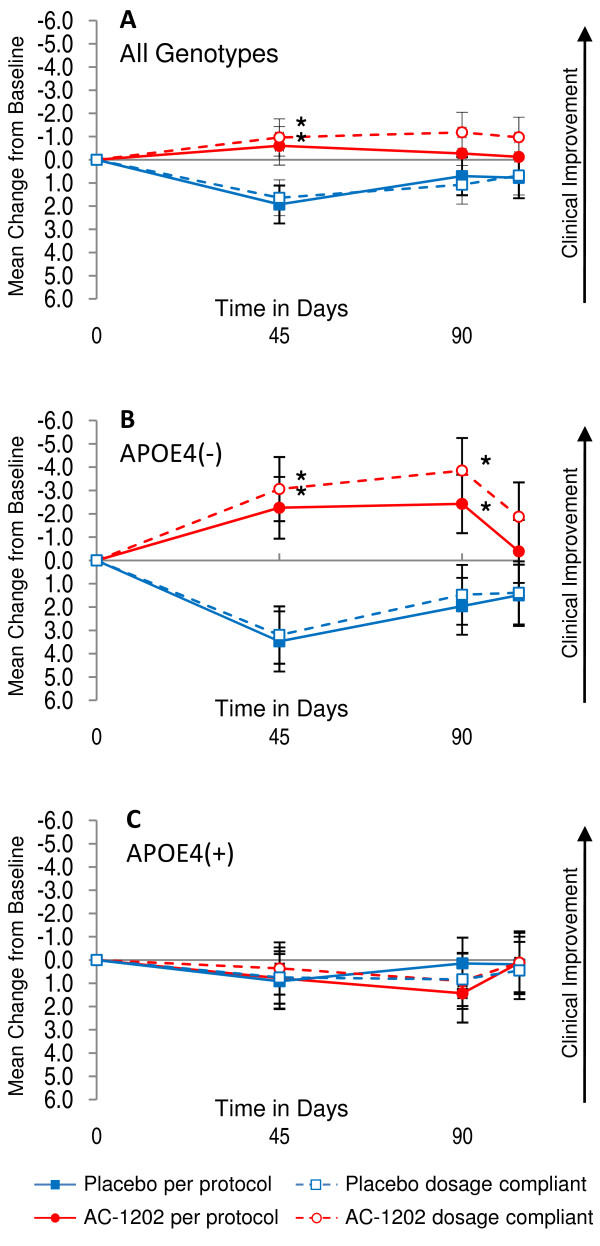
**Mean change in ADAS-Cog scores from Baseline in per protocol and dosage compliant populations without LOCF and stratified by APOE4 carriage status**. Y axis is change from Baseline. X axis is time in days. Solid red circles and lines represent per protocol subjects taking AC-1202. Solid blue squares and lines represent per protocol subjects taking Placebo. Open red circles and dashed lines represent dosage compliant subjects taking AC-1202. Open blue squares and dashed lines represent dosage compliant subjects taking Placebo. Error bars represent standard error of the mean. Asterisks (*) indicate a significant (p-value < 0.05) difference in mean change from Baseline between AC-1202 and Placebo. **A) **Per protocol and dosage compliant subjects administered AC-1202 regardless of genotype, both cohorts demonstrate a significant difference from Placebo at Day 45. **B) **Per protocol and dosage compliant subjects lacking the APOE4 allele (APOE4(-)) and administered AC-1202 demonstrate a significant difference from Placebo at both Days 45 and 90. **C) **Per protocol and dosage compliant subjects carrying the APOE4 allele (APOE4(+)) do not differ at any time point. For number of subjects, confidence intervals, and p-values, see Tables 6 and 7.

In the per protocol population (N = 91), there was a significant effect between groups in change from Baseline in ADAS-Cog scores, regardless of genotype, on Day 45 (p = 0.0324) but not at Day 90 (p = 0.192) (Figure 4A, Table 6). As with the ITT population, when this group was stratified by APOE4 status, a significant effect in ADAS-Cog was observed. Within the per protocol group, E4(-) subjects receiving AC-1202 were significantly different from Placebo in ADAS-Cog change from Baseline at Day 45 (5.73 point difference, p = 0.0027) and at Day 90 (4.39 point difference, p = 0.0143), but not after Washout (p = 0.321) (Figure 4B, Table 6). There were no significant effects of AC-1202 in the per protocol E4(+) subjects in any cognitive outcome measure (Figure 4C, Table 6).

**Table 6:** Efficacy by visit and genotype in the per protocol population*

**Group; N**	**AC-1202**	**Placebo**	**Difference (95% CI)**	**p-value**
	**Day 45 Mean Change From Baseline**			

**ADAS-Cog**				
PP; 45AC, 46PL	-0.600	1.927	2.53(0.22, 4.84)	0.0324
APOE4(-); 18AC, 19PL	-2.259	3.472	5.73(2.05, 9.41)	0.0027
APOE4(+); 20AC, 23PL	0.783	0.913	0.13(-3.29, 3.55)	0.9400
**MMSE**				
PP; 45AC, 46PL	0.378	-0.217	0.60(-0.55, 1.74)	0.3060
APOE4(-); 18AC, 19PL	0.000	0.316	0.32(-1.44, 2.07)	0.7207
APOE4(+); 20AC, 23PL	0.350	-0.826	1.18(-0.45, 2.81)	0.1546
**CGIC^†^**				
PP; 45AC, 46PL	4.00	4.26	0.26(-0.24, 0.76)	0.3040
APOE4(-); 18AC, 19PL	3.94	4.89	0.95(0.20, 1.70)	0.0142
APOE4(+); 20AC, 23PL	4.15	3.61	0.54(-0.16, 1.24)	0.1283

	**Day 90 Mean Change From Baseline**			

**ADAS-Cog**				
PP; 45AC, 46PL	-0.563	0.956	1.52(-0.78, 3.82)	0.1923
APOE4(-); 18AC, 19PL	-2.426	1.963	4.39(0.90, 7.87)	0.0143
APOE4(+); 20AC, 23PL	1.433	0.145	-1.29(-4.53, 1.95)	0.4307
**MMSE**				
PP; 45AC, 46PL	-0.261	-0.178	0.08(-1.14, 1.30)	0.8925
APOE4(-); 18AC, 19PL	-0.056	0.684	0.74(-1.20, 2.68)	0.4502
APOE4(+); 20AC, 23PL	-0.350	-0.913	0.56(-1.24, 2.37)	0.5362
**CGIC^†^**				
PP; 45AC, 46PL	4.31	4.61	0.29(-0.28, 0.87)	0.3109
APOE4(-); 18AC, 19PL	3.83	4.58	0.75(-0.15, 1.64)	0.1006
APOE4(+); 20AC, 23PL	4.65	4.43	0.22(-0.62, 1.04)	0.6072

	**Day 104 Mean Change From Baseline**			

**ADAS-Cog**				
PP; 45AC, 46PL	-0.274	0.704	0.98(-1.35, 3.30)	0.4055
APOE4(-); 18AC, 19PL	-0.389	1.495	1.88(-1.87, 5.64)	0.3206
APOE4(+); 20AC, 23PL	0.117	0.188	0.07(-3.42, 3.56)	0.9674
**MMSE**				
PP; 45AC, 46PL	0.444	-0.587	1.03(-0.18, 2.24)	0.0941
APOE4(-); 18AC, 19PL	-0.111	0.158	0.27(-1.65, 2.19)	0.7813
APOE4(+); 20AC, 23PL	1.000	-1.130	2.13(0.34, 3.92)	0.0201
**CGIC^†^**				
PP; 45AC, 46PL	4.64	4.78	0.14(-0.44, 0.72)	0.6368
APOE4(-); 18AC, 19PL	4.22	4.89	0.67(-0.22, 1.56)	0.1359
APOE4(+); 20AC, 23PL	4.80	4.52	0.28(-0.55, 1.10)	0.5043

PP = per protocol; AC = AC-1202; PL = Placebo.

*2 way ANOVA calculated using PROC GLM Type 3 SS. P-values of differences of the type 3 SS LSMEANS.

^†^CGIC Mean Total scores at Days 45, 90 and 104.

#### MMSE

In general there were no significant changes in MMSE scores from Baseline in the per protocol population between AC-1202 and Placebo groups. Among E4(+) subjects, changes in MMSE score were significantly different on Day 104. On Day 104, mean change from baseline in MMSE scores among E4(+) subjects improved one point in the AC-1202 group, while the Placebo group declined on average -1.13 points (p = 0.0201).

#### CGIC

Among E4(-) subjects in the per protocol subgroup, those administered AC-1202 showed significantly lower CGIC scores at Day 45 (p = 0.0142) compared with Placebo. CGIC scores at Day 90 and Day 104 in the AC-1202 group were not significantly different from Placebo (Day 90, p = 0.1006; Day 104, p = 0.1359). Among E4(+) participants, average scores on CGIC were similar in both groups at all time points (Table 6).

### Dosage Compliant subjects

To examine the effect of compliance to the dosing schedule on cognitive outcomes, an analysis was completed on dosage compliant subjects. Dosage compliant subjects were defined as those that reported consuming a total cumulative dose of at least 80% of the total intended dose. Among the 77 AC-1202 subjects, 46 were classified as compliant and 31 were not. Among the 63 Placebo subjects, 50 were classified as compliant and 13 were not. Analysis of cognitive outcomes in the compliant population was done using the patients scores at each visit, LOCF was not used in analysis of test scores for compliant subjects. Therefore, this analysis provides insight into changes in cognitive function among subjects who took the investigational product and does not use any imputed data.

#### ADAS-Cog

Among the dosage compliant population, there was a significant difference in change from Baseline in ADAS-Cog scores between those administered AC-1202 compared to those administered Placebo on Day 45 (2.60 point difference; p = 0.0215), but not on Day 90 (2.26 point difference; p = 0.064) or Day 104 (1.68 point difference; p = 0.143) (Figure 4A, Table 7). Among E4(-) dosage compliant subjects, there was a significant difference in change from Baseline in ADAS-Cog scores between those administered AC-1202 compared to those administered Placebo on Day 45 (6.26 point difference; p = 0.001) and at Day 90 (5.33 point difference; p = 0.006), but not at Day 104 (3.26 point difference; p = 0.107) (Figure 4B, Table 7). Among E4(+) compliant subjects, there was no significant difference in change from Baseline in ADAS-Cog scores between those administered AC-1202 compared to those administered Placebo at any time point (Figure 4C, Table 7).

**Table 7:** Efficacy by visit and genotype in the dosage compliant population*

**Group; N**	**AC-1202**	**Placebo**	**Difference (95% CI)**	**p-value**
	**Day 45 Mean Change From Baseline**			

**ADAS-Cog**				
DC; 46AC, 50PL	-0.964	1.639	2.60(0.39, 4.81)	0.0215
APOE4(-); 16AC, 20PL	-3.063	3.198	6.26(2.59, 9.94)	0.0011
APOE4(+); 24AC, 24PL	0.361	0.750	0.39(-2.77, 3.55)	0.8073
**MMSE**				
DC; 46AC, 50PL	0.326	-0.220	0.55(-0.57, 1.66)	0.3342
APOE4(-); 16AC, 20PL	-0.313	0.350	0.66(-1.12, 2.45)	0.4622
APOE4(+); 24AC, 24PL	0.500	-0.917	1.42(-0.12, 2.95)	0.0701
**CGIC^†^**				
DC; 44AC, 48PL	4.114	4.313	0.20(-0.32, 0.72)	0.4481
APOE4(-); 16AC, 19PL	4.063	4.895	0.83(0.00, 1.66)	0.0500
APOE4(+); 22AC, 23PL	4.182	3.652	0.53(-0.20, 1.26)	0.1533

	**Day 90 Mean Change From Baseline**			

**ADAS-Cog**				
DC; 44AC, 48PL	-1.182	1.076	2.26(-0.14, 4.65)	0.0641
APOE4(-); 16AC, 19PL	-3.854	1.472	5.33(1.55, 9.11)	0.0063
APOE4(+); 22AC, 24PL	0.909	0.833	0.08(-3.21, 3.36)	0.9635
**MMSE**				
DC; 44AC, 48PL	-0.136	-0.271	0.13(-1.1, 1.36)	0.8275
APOE4(-); 16AC, 19PL	-0.125	0.789	0.91(-1.09, 2.92)	0.3656
APOE4(+); 22AC, 24PL	-0.136	-1.083	0.95(-0.79, 2.69)	0.2820
**CGIC^†^**				
DC; 43AC, 48PL	4.535	4.521	0.01(-0.56, 0.59)	0.9613
APOE4(-); 16AC, 19PL	4.125	4.474	0.35(-0.59, 1.29)	0.4613
APOE4(+); 21AC, 24PL	4.714	4.375	0.34(-0.49, 1.17)	0.4158

	**Day 104 Mean Change From Baseline**			

**ADAS-Cog**				
DC; 43AC, 47PL	-0.969	0.682	1.65(-0.75, 4.05)	0.1751
APOE4(-); 16AC, 19PL	-1.875	1.389	3.26(-0.72, 7.25)	0.1070
APOE4(+); 21AC, 23PL	0.111	0.449	0.34(-3.21, 3.88)	0.8498
**MMSE**				
DC; 43AC, 47PL	0.186	-0.766	0.95(-0.48, 2.38)	0.1901
APOE4(-); 16AC, 19PL	-0.313	0.000	0.31(-2.03, 2.66)	0.7916
APOE4(+); 21AC, 23PL	0.524	-1.565	2.09(0.00, 4.18)	0.0499
**CGIC^†^**				
DC; 42AC, 46PL	4.667	4.804	0.14(-0.42, 0.70)	0.6248
APOE4(-); 15AC, 19PL	4.267	4.895	0.63(-0.24, 1.50)	0.1547
APOE4(+); 21AC, 22PL	4.857	4.455	0.40(-0.37, 1.17)	0.3002

DC = Dosage Compliant; AC = AC-1202; PL = Placebo.

*2 way ANOVA calculated using PROC GLM Type 3 SS. P-values of differences of the type 3 SS LSMEANS.

^†^CGIC Mean Total scores at Days 45, 90 and 104.

Note, since this table uses non-imputed data, the number of subjects varies by Visit.

#### MMSE

In general there were no significant changes in MMSE scores from Baseline in the dosage compliant population between AC-1202 and Placebo groups. Among E4(+) subjects, changes in MMSE score were significantly different between groups on Day 104. On Day 104, mean change from baseline in MMSE scores among E4(+) subjects improved on average 0.524 points in the AC-1202 group, while the Placebo group declined on average -1.565 points (p = 0.0499) (Table 7).

#### CGIC

Among E4(-) subjects in the dosage compliant subgroup, those administered AC-1202 showed significantly lower CGIC scores at Day 45 (p = 0.05) compared with Placebo. CGIC scores at Day 90 and Day 104 in the AC-1202 group were not significantly different from Placebo (Day 90, p = 0.4613; Day 104, p = 0.1547). Among E4(+) participants in the dosage compliant subgroup, average scores on CGIC were similar to Placebo at all time points (Table 7).

### Total Dose and Change in ADAS-Cog at Day 90

**Figure 5:**
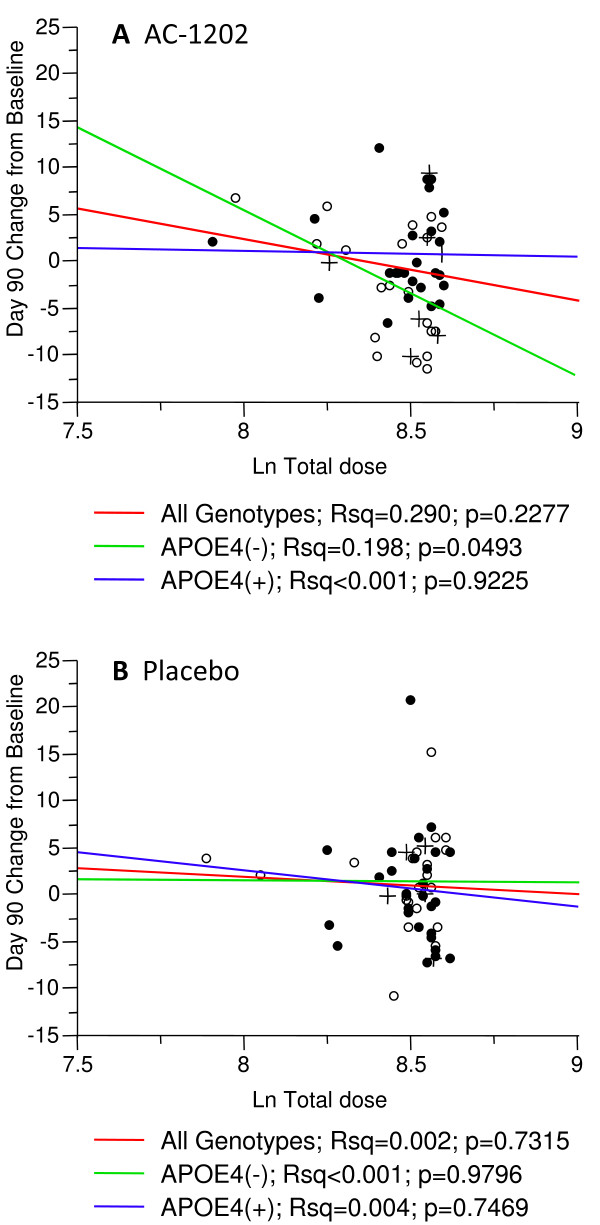
**Linear fit of change from Baseline at Day 90 in ADAS-Cog score and log transformed total dosage stratified by APOE4 carriage status**. Only reported ADAS-Cog scores were used; no data was imputed. Solid circles represent APOE4(+) subjects, open circles represent APOE4(-) subjects. Crosses represent non-dosage compliant subjects. Red line indicates linear fit for all genotypes, green line for APOE4(-) subjects, and blue line for APOE4(+) subjects. **A) **Among subjects taking AC-1202, a significant correlation was found in APOE4(-) subjects in change from Baseline at Day 90 in ADAS-Cog score and log transformed total dosage. **B) **Among subjects taking Placebo, no significant correlations were found in change from Baseline at Day 90 in ADAS-Cog score and log transformed total dosage.

To examine the effect of total dosage on cognitive performance, an analysis was completed correlating the change from Baseline in ADAS-Cog scores at Day 90 with total dose reported by each patient. This analysis did not use LOCF, only actual reported scores were used. Among subjects of any genotype receiving AC-1202, there was no significant correlation between total dose and change in ADAS-Cog at Day 90 (p = 0.2277) (Figure 5A). Among E4(-) subjects receiving AC-1202, there was a significant correlation between total dose and change in ADAS-Cog at Day 90 (p = 0.0493) (Figure 5A). In E4(-) participants, larger total doses correlated with improved performance on the ADAS-Cog test (lower scores) (Figure 5A). No significant correlations were found in E4(+) subjects receiving AC-1202 (p = 0.9225), or in any of the groups receiving Placebo (Figure 5B).

**Figure 6:**
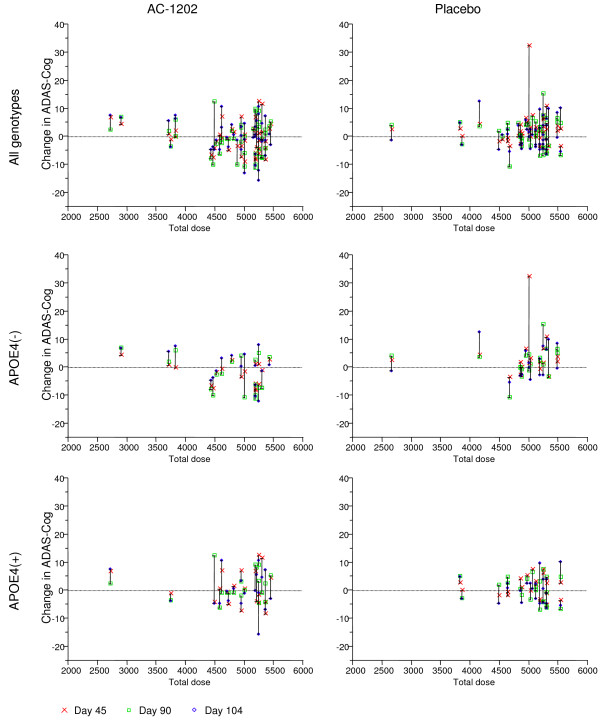
**Plot of change in ADAS-Cog and total dose administered for each of the per protocol participants**. Y axis is change in ADAS-Cog score from Baseline. X axis is total dose in grams. Each subject is represented by three symbols. A red x represents the change from Baseline score at Day 45. A green square represents the change from Baseline at Day 90. A blue diamond represents the change from Baseline at Day 104. APOE4(-) subjects who received more than 4000 grams generally improved in ADAS-Cog score.

A plot of total dose consumed by each patient in the per protocol population versus change in ADAS-Cog scores at Days 45, 90 and 104 is shown in Figure 6. In general, E4(-) subjects who consumed greater than 4000 grams showed improvement in ADAS-Cog.

### Serum BHB and ADAS-Cog

**Figure 7:**
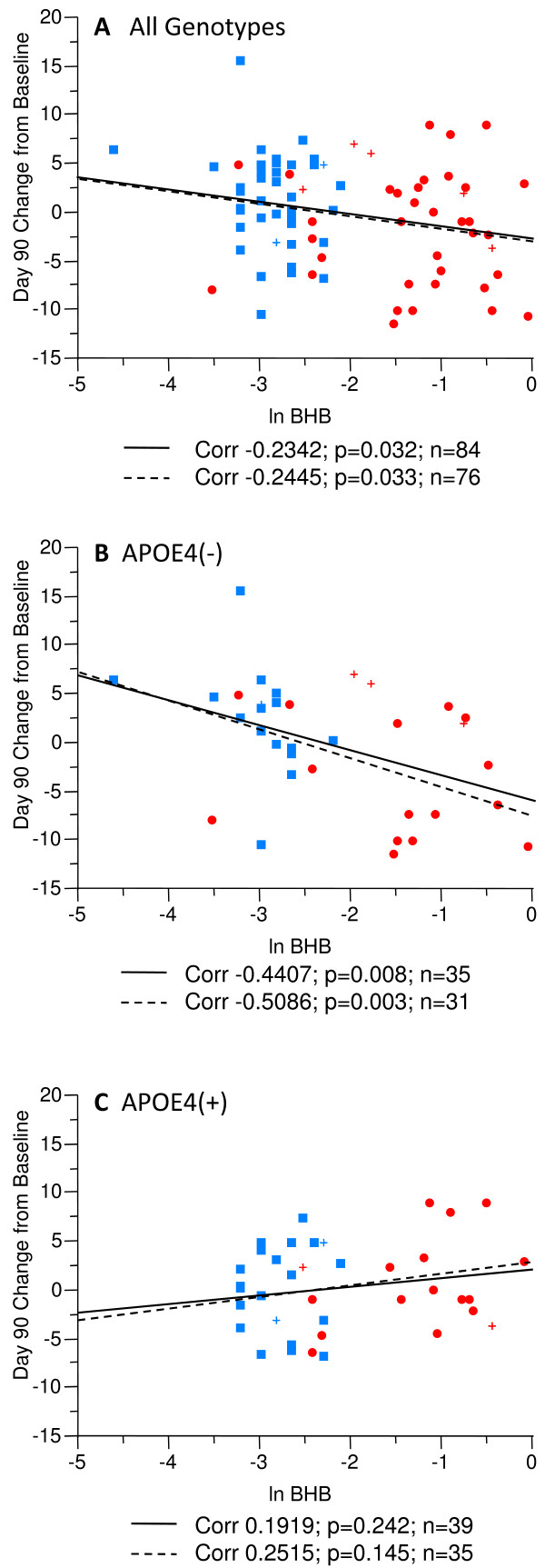
**Linear fit of change from Baseline at Day 90 in ADAS-Cog score and log transformed post-dose serum BHB levels in the per protocol population stratified by APOE4 carriage status**. Red symbols represent subjects taking AC-1202. Blue symbols represent subjects taking Placebo. Circles and squares indicate dosage compliant subjects, crosses represent non-compliant subjects. The solid lines represent the linear fit in the per protocol population. The dashed lines represent the linear fit in the dosage compliant sub-population of the per protocol population. **A) **A significant correlation was found in the per protocol population between serum BHB levels and change in ADAS-Cog on Day 90. **B) **A significant correlation was found in E4(-) per protocol subjects between serum BHB levels and change in ADAS-Cog on Day 90. **C) **No significant correlation was found in E4(+) per protocol subjects between serum BHB levels and change in ADAS-Cog on Day 90. In each case the linear fit of the dosage compliant subgroup is very similar to the overall per protocol population.

To examine the effect of elevated BHB on cognitive performance, an analysis was completed on subjects who had BHB blood draws and ADAS-Cog testing on the same day. Figure 7 illustrates a correlation between change in ADAS-Cog scores and serum BHB concentration at Day 90 in the per protocol population. A significant correlation between BHB plasma levels and change in ADAS-Cog scores was observed in the overall population irrespective of genotype (p = 0.032) (Figure 7A) as well as in E4(-) subjects (p = 0.008) (Figure 7B). Higher serum BHB concentrations correlated with lower ADAS-Cog scores. No correlation between ADAS-Cog scores and BHB plasma levels was observed in E4(+) subjects (Figure 7C). An additional analysis was done removing the non-dosage compliant subjects, yet this did not significantly alter the outcomes. When non-dosage compliant subjects are removed from the analyses, a significant correlation was maintained in both the overall population (p = 0.033) and among E4(-) participants (p = 0.003) (Figure 7A).

### Safety Analyses

Safety assessments were performed for all subjects enrolled in the study. Gastrointestinal (GI) events were the most frequently-reported adverse events (AEs) in both groups. A total of 48.8% of AC-1202 subjects and 27.3% of Placebo subjects experienced at least one GI AE. Diarrhea, occurring in 24.4% of AC-1202 and 13.6% of Placebo subjects, was the most frequently reported AE in both groups. The proportion of subjects within each group discontinuing the study for AEs was notably higher in the AC-1202 group compared with Placebo (23.3% AC; 6.1% PL). After product mixing instructions were changed, the severity and the number of subjects discontinuing the study for GI AEs notably declined. Prior to the change in mixing instructions, 3 of 31 (9.7%) AC-1202 subjects experienced severe diarrhea compared with 0 of 27 Placebo subjects. After the mixing instruction change, the rate of severe diarrhea reported in AC-1202 group declined to 2 of 64 (3.1%). Likewise, the rate of study discontinuations in the AC-1202 group for any type of GI-related event declined from 7 of 31 (22.6%) before the change, to 8 of 64 (12.5%) after the change.

In general, no significant differences between groups were observed for changes in serum chemistry and hematology values, vital signs, or electrocardiograms. Mean values in renal function tests increased from screening in the AC-1202 group, however, the increases did not exceed 2.5 × upper limit of normal in any of the subjects and these changes were not considered clinically significant by the investigators. Five of seven AC-1202 subjects with notable elevations in BUN, creatinine, or uric acid levels had abnormally high values at Screening that were further increased at Day 104. Among these subjects, three were noted to have apparent urinary tract infections at study enrollment, and one had a history of renal failure requiring dialysis prior to enrollment. Furthermore, all of the subjects with significant renal function test abnormalities were noted to have BUN/creatinine ratios >15, a likely indication of dehydration. While the increases in these lab tests in the AC-1202 group appear to be related to either pre-existing co-morbidities or to dehydration, a relation to study investigational product cannot be entirely ruled out. APOE4 positive and negative subjects receiving AC-1202 experienced similar rates of adverse events, and neither the frequency nor the characteristics of these AEs appeared to be associated with the presence or absence of the APOE4 allele.

## Discussion

Ketogenic diets have been used for over 50 years in a variety of CNS conditions. For example, in a 3-month randomized controlled trial, a ketogenic diet resulted in an almost 75% reduction of seizure frequency in childhood epilepsy [33]. The long-term tolerability and effects of ketogenic diets have also been previously reported [17]. In addition, there are preclinical data on the beneficial effects of intermittent fasting, caloric restriction and ketogenic diets on brain amyloid deposition and toxicity (for review see [22]).

Ketogenic diets result in many changes, other than simply elevating circulating ketone body levels, which may confer neuroprotection. For example, ketogenic diets have been found to increase levels and activity of uncoupling proteins [34]. To address whether administration of ketone bodies alone are neuroprotective, several studies have examined if infusion of BHB would protect cells from a variety of cytotoxic agents and conditions. Infusion of BHB was found to protect rodents from 1-methyl-4-phenyl-1,2,3,6-tetrahydropyridine (MPTP) [35], hypoxia [20,36], traumatic brain injury [37] and glutamate toxicity [38]. Such studies suggest that ketone bodies alone offer a possible therapeutic intervention under several conditions (for review see [21]).

In this study, we examined if chronic induction of mild ketosis would be beneficial to patients with mild to moderate AD. Chronic induction of ketosis in AD patients could be achieved by compliance to a ketogenic diet. However, ketogenic diets require strict adherence to low carbohydrate intake. Compliance to low carbohydrate intake may be difficult for Alzheimer's patients due to the well documented shift in food preference toward sweet, carbohydrate-rich foods [25,39]. We took advantage of the unique properties of MCTs to induce ketosis without the need for dietary change. AC-1202 successfully induced mild ketosis in AD subjects. Administration of AC-1202 at 10 grams (1/2 recommended amount) at Baseline significantly elevated average serum BHB levels by 157%, 2 hours post-dose. Administration of 20 grams of AC-1202 (full recommended amount) significantly elevated average serum BHB levels by 330% on Day 45 and by 401% on Day 90. In contrast, in the Placebo group, average serum BHB levels declined between pre- and post-dose sampling at each study visit, possibly due to suppression of endogenous BHB production after eating a carbohydrate-rich breakfast. Notably, AC-1202 was able to elevate serum BHB even when the subjects ate breakfast. These findings are consistent with the ketogenic properties of MCTs [30].

AC-1202 is an MCT composed almost entirely of C8:0 fatty acids. It is possible that some beneficial action could be attributed to the C8 fatty acids rather than ketone bodies. Several reports have implicated MCTs in increasing fatty acid oxidation with possible roles in weight loss [40,41]. However, in general, very little C8 reaches the blood stream after ingestion of an MCT. Medium chain triglycerides containing C8 fatty acids undergo complete hydrolysis in the gut lumen. The released C8 are poor substrates for esterification and instead are transported by the portal vein directly to the liver. Within the liver, C8 fatty acids undergo obligate oxidation. Thus, very little C8 makes it in to circulation (for overview see [29,30,42]). In studies in humans dosed with C8 containing MCTs, very little free C8 is found in aterial blood [43]. While we cannot entirely rule out the role of C8 fatty acids in mediating some of the cognitive effects seen in the study, the correlation of cognitive performance with circulating BHB suggests that ketosis plays a prominent role in MCT therapy.

The levels of ketosis obtained with AC-1202 were mild and similar to those seen in the early phases of very low carbohydrate diets, and much lower than levels found during starvation or diabetic ketoacidosis. Very low carbohydrate diets have been examined mainly for weight loss and management of type II diabetes (for review see [44]). In studies of low carbohydrate diets, average levels of BHB after 2 weeks range from 0.4 mM to 0.65 mM, and these levels frequently decrease over time and may return to Baseline after 10 to 12 weeks [45-48]. Ketogenic diets differ from a low carbohydrate diets by imposing stricter limitations on protein intake, and higher, more sustained levels of BHB (above 1 mM) have been reported [49,50]. As a comparison, much higher levels of serum BHB are found during 5–6 weeks of starvation (4–8 mM) [51] and in cases of diabetic ketoacidosis (9–10 mM) [52] (Table 8). AC-1202 induced transient increases of ketosis that reached average levels of 0.3 to 0.4 mM in the 2 hour post-dose sample on Days 45 and 90. The positive cognitive effects noted in E4(-) subjects suggests that higher levels of ketosis may not be required in AD patients to produce beneficial outcomes, and that safe, mild elevations in BHB can be effective.

**Table 8:** Levels of BHB associated with AC-1202, fasting, and dietary regimens

**Intervention**	**Average BHB** **levels, mM**	**Notes**	**Reference**
12 hour fast	0.08 – 0.1	Fasting level on typical diet	This study, [47,48]

AC-1202 (20 grams)	0.36	2 hour post-dose levels	This study

Low carbohydrate diet	0.4 – 0.65	Low carbohydrate diets ranging from 4–10% carbohydrate	[45-48]

Ketogenic diet	0.3 – 1.6	Ketogenic diets given to children with refractive epilepsy	[49,50]

Starvation	4 – 8	5–6 weeks of complete starvation	[51]

Diabetic ketoacidosis	9 – 10	Insulin deficiency and elevated levels of counter-regulatory hormones	[52]

While the direct effects of ketone bodies on AD metabolism have not been definitively shown, preclinical studies provide clues as to the possible mechanism of action. The induction of mild ketosis by AC-1202 in an aged dog model was found to improve respiration rates in the parietal regions of the brain, by improving mitochondrial function and reducing mitochondrial oxidative damage [53]. This is consistent with studies demonstrating improved mitochondrial function in rat hearts perfused with BHB [15]. In addition, Kashiwaya et. al. tested the ability of BHB to protect cultured hippocampal neurons from the toxic effects of Aβ42. Four millimolar BHB was found to significantly protect the neurons from Aβ42 [23]. The proposed mechanism of protection was improved mitochondrial efficiency. In cell culture studies, incubation with ketone bodies reduced need for glycolysis, increased metabolites in the first third of the TCA cycle, and increased the redox potential of the NAD/NADH couple [15]. In addition, exposure to BHB has been reported to increase autophagy [54] and activate HIF-1(alpha) [55]. Further experimentation will be required to understand the precise mechanism whereby AC-1202 improves cognitive performance in AD patients. Yet, the rapid response seen in an earlier study [31], suggests that improvement in cellular metabolism plays a prominent role. For a discussion of the role of ketone bodies in Alzheimer's disease see Henderson [14].

While the cognitive effects were not significant in the overall sample, a pre-defined examination of cognitive effects stratified by genotype yielded significant effects in E4(-) participants. The ADAS-Cog difference between AC-1202 and Placebo of 4.77 points at Day 45 and 3.36 points at Day 90 is notable given that many of the subjects in both groups were already receiving cholinesterase inhibitors and/or memantine. After the two week Washout (Day 104), there was no difference between groups. E4(+) subjects did not differ between groups at any time point.

Near the end of the study, newly enrolled participants were intentionally assigned to AC-1202 or Placebo groups by an independent monitor to balance the number of subjects who completed the study in each group. Since this intentional assignment may have introduced bias into the study, we conducted an analysis of "randomized only" subjects. The analysis of the randomized only subjects mirrors both the ITT w/LOCF population, and the per protocol population. In each analysis, there is no significant effect in the primary outcomes. Yet, in each analysis, significant effects in change from Baseline in ADAS-Cog scores compared to Placebo were found in APOE4(-) subjects, at both Day 45 and Day 90. In addition, in each case, the significant effects on APOE4(-) subjects is lost after the two week washout (Day 104).

Intention-to-treat w/LOCF analysis is beneficial in that it captures data from participants who may not have tolerated the complete study regimen. Yet, since AD is a progressive disease, a reliance on the ITT w/LOCF analysis may have biased the results toward AC-1202, given that the AC-1202 group experienced a higher dropout rate compared to the Placebo group. To address this concern, we conducted an analysis of "per protocol" subjects. Per protocol subjects were defined as subjects who completed all study visits and efficacy measures and had no values carried forward. Despite the relatively small numbers, the results of the per protocol analyses mirror the ITT w/LOCF results, with better results seen in the per protocol population. Among all per protocol subjects, a 2.53 point difference between groups in change from Baseline in ADAS-Cog was found at Day 45 compared to a 1.91 point difference in the ITT population. As with the ITT w/LOCF analysis, better efficacy was found in the per protocol E4(-) sub-population. On Day 45, a 5.73 point difference between groups in change from Baseline in ADAS-Cog was found in the E4(-) per protocol population compared to a 3.05 point difference in the E4(-) ITT population. On Day 90, a 4.39 point difference between groups in change from Baseline in ADAS-Cog was found in the E4(-) per protocol population compared to a 3.36 point difference in the E4(-) ITT population. The significant differences found between AC-1202 and Placebo groups in the per protocol analysis suggest that the positive efficacy results are not simply due to the use of imputed data. The consistent finding of an effect in E4(-) subjects in the total population, per protocol, and randomized only subgroups, suggests that this result is not due to the introduction of bias.

**Figure 8:**
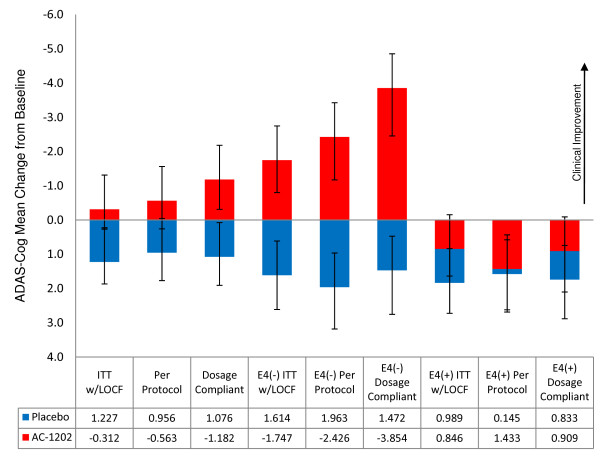
**Summary graph of mean change from Baseline at Day 90 for ITT w/LOCF, per protocol and dosage compliant groups stratified by APOE4 carriage status**. Red columns represent subjects receiving AC-1202. Blue columns represent subjects receiving Placebo. Error bars represent standard error of the mean. Table displays mean change from Baseline for each group. Mean changes from Baseline was largest in APOE4(-) subjects who were dosage compliant.

If AC-1202 is producing a positive effect in E4(-) subjects, it is reasonable to hypothesize that such an effect would be most notable in subjects who complied to the dosing schedule and actually took a substantial percentage of the investigational product. This hypothesis is supported by the analysis of dosage compliant subjects and the correlation between total dosage and improvement in ADAS-Cog at Day 90. The subjects that demonstrated the most response to therapy at Day 90 were E4(-) subjects who were dosage compliant (Figure 8). Among E4(-) subjects, mean change from Baseline in ADAS-Cog score at Day 90 (independent of Placebo) was -1.7 points in the ITT w/LOCF population, -2.4 points in the per protocol population, and -3.9 points in the dosage compliant population. Dosage compliant subjects also performed better at Day 45 (-3.1 points) than either the per protocol population (-2.3 points) or the ITT w/LOCF population (-1.7 points). In addition, among E4(-) subjects, there was a significant correlation between total dose administered and change in ADAS-Cog score from Baseline at Day 90.

The positive effects of AC-1202 in E4(-) subjects is further supported by analysis of serum BHB levels and cognitive performance. AC-1202 resulted in significant elevation of serum BHB relative to Placebo at all study visits when investigational product was administered. In addition, a correlation between circulating BHB levels at the two-hour time point and improvement in ADAS-Cog score was noted in E4(-) subjects at Day 90. No significant correlation was found in E4(+) participants. Hence, higher levels of ketosis appear to confer greater benefit in the E4(-) group.

A common metric for clinically significant changes in AD trials is a 4 point change in ADAS-Cog after 6 months. This is frequently represented as the percent of subjects in each group who achieved this benchmark [56,57]. In the present study, participants were only on therapy for 3 months, yet many of the subjects reached this level of improvement. Among E4(-) subjects receiving AC-1202, 31% (9/29) experienced a -4 point or greater improvement compared to 7.7% (2/26) in the Placebo group at Day 90. Among E4(-) subjects receiving AC-1202 who were also dosage compliant, 50% (8/16)) experienced a -4 point or greater improvement compared to 10% (2/20) in the Placebo group at Day 90.

We can only speculate on the mechanisms underlying the genotype-specific effects seen in this study, but there are reasons to suggest such an effect is not spurious. For example, E4(-) AD subjects seem to have greater relative benefits associated with some other therapies, such as infusion with glucose and insulin [58], nasal insulin, [59] or the insulin sensitizing agent rosiglitazone [60]. One hypothesis is that there may be lower mitochondrial enzyme function in E4(+) versus E4(-) as noted in AD brain tissue samples [61,62]. Reduced mitochondrial function may inhibit the ability of E4(+) participants to utilize ketone bodies and this may explain the apparent unresponsiveness to AC-1202 reported here.

An alternative explanation may be a differential insulin sensitivity of AD subjects based on APOE genotype [10,60,63]. Ketone bodies are transported into the brain by monocarboxylate transporters [64]. Levels of monocarboxylate transporters in the microvasculature are known to be low in adult mammals, yet elevated in diabetes and in other conditions where insulin resistance occurs [65]. The milder insulin resistance in E4(-) AD subjects may allow them to more efficiently import ketone bodies into the brain and hence respond to AC-1202. Such a model is consistent with the observation that serum concentrations of BHB correlated with improvement in cognitive performance in E4(-) subjects, but not in E4(+) subjects. However, these models remain speculative and further experimentation will be required to confirm these differences and underlying mechanisms.

The rapid effects of induced ketosis in an earlier study [31] and the loss of statistically significant differences in ADAS-Cog scores after the two week washout, suggests that AC-1202 may function to improve neuronal or glial metabolism in the presence of AD pathology, but may not slow the disease process (at least in a 3 month study). These results suggest that improvement in cognition may be feasible by mechanisms that do not address more typical targets, such as amyloid or tau. Improvement in mitochondrial efficiency, or activation of protective pathways, may provide a viable means to address AD. In support of this view, in animal models of AD, positive cognitive outcomes have been reported by interventions that do not lower amyloid or tau levels. For example, Halagappa et. al. demonstrated in a triple transgenic mouse model of AD that an intermittent fasting regime (which may elevate ketone bodies) produced cognitive benefits without affecting levels of Aβ or Tau [66].

### Limitations

As this is a new area of AD research, our findings must be interpreted in that context. Participants administered Placebo demonstrated an unusual degree of worsening in ADAS-Cog scores at Day 45. The magnitude of this change is somewhat unexpected, although other studies have reported similar rates of decline [67,68]. The lack of strong efficacy in MMSE and CGIC may be due to relative insensitivity of these tests, the small number of subjects, and/or the short duration of the trial. In addition, the reported deviations in the CGIC assessment scales cautions against over-interpretation of these results. The relatively high drop outs due to GI effects with our initial dosing regimen led to an intentional assignment by an independent monitor of a small number of subjects to the Placebo and AC-1202 groups, yet this did not appear to influence the overall outcome. It is possible that GI side effects might have biased outcomes in some subtle manner, although it must be noted that high rates of GI side effects are also noted in cholinesterase inhibitor trials. Group randomization was not stratified by APOE genotype, yet distribution of APOE4 alleles was similar between groups. Lastly, since this was a 3-month trial, this study cannot address the safety and efficacy of longer periods of administration.

## Conclusion

Due to the unique mechanism of action of AC-1202, participants were allowed to continue on stable concomitant AD treatments. Approximately 80% of the subjects in both groups were on one form of AD therapy. Despite being tested in a non-naïve population, AC-1202 resulted in significant improvement in the ADAS-Cog test in E4(-) AD patients relative to Placebo. The GI side effects were predictable, and data from this trial will allow further optimization of the administration of AC-1202 to minimize such events. Therefore, chronic induction of ketosis may offer a novel strategy for AD that can be used with current therapies.

## List of abbreviations

ACA: Acetoacetate; AD: Alzheimer's disease; ADAS-Cog: AD Assessment Scale-Cognitive subscale; ADCS-CGIC: AD Cooperative Study – Clinical Global Impression of Change; AEs: Adverse Events; BHB: β-hydroxybutyrate; ITT: Intention-to-treat; LOCF: Last Observation Carried Forward; MMSE: Mini Mental State Exam.

## Statement of Competing interests

Some authors may benefit from this publication. Authors SH, LB, JV, FG and LC are, or were, employees of the sponsor of this trial, Accera Inc. Accera funded the study, designed the protocol, and either conducted or commissioned the data analysis and interpretation. Accera was responsible for: collection, analysis, and interpretation of data; writing of the paper; and decision to submit it for publication.

SH has very minor stock ownership in Accera.

SH is the sole inventor of one issued patent (US 6835750) entitled: Use of medium chain triglycerides for the treatment and prevention of Alzheimer's disease and other diseases resulting from reduced neuronal metabolism II. SH and Accera have other published pending patent applications in this area: US 2002/0006959 A1, US 2003/0059824 A1, US 2006/0122270 A1, US 2008/0009467 A1, US 2006/0252775 A1, US 2007/0135376 A1, US 2008/0287372 A1, US 2007/0179197 A1.

SH, LB, JV and LC are, or were, employees of Accera and, as such, have the option to purchase stock in Accera.

Accera intends to market a product for Alzheimer's disease using AC-1202 called Axona™.

## Authors' contributions

Author SH is the primary author of the paper and designed the basis of the scientific approach. JJ was an outside statistical consultant contracted by Accera, Inc to perform statistical analysis of the clinical trial data. FG was contracted by Accera, Inc to perform quality control and data verification of the clinical trial data. LB, JV, FG, JJ, and LC collected data, performed quality control, analyzed data, and revised the manuscript during its preparation. All authors have read and approved the final manuscript.
